# An essential role of IκBζ in the transcriptional program in Th17 development

**DOI:** 10.1186/ar3691

**Published:** 2012-02-09

**Authors:** Kazuo Okamoto, Masatsugu Oh-hora, Hiroshi Takayanagi

**Affiliations:** 1 Department of Cell Signaling, Tokyo Medical and Dental University, Tokyo, Japan. 2GCOE Program, Tokyo, Japan. 3JST, ERATO, Takayanagi Osteonetwork Project, Tokyo, Japan

## 

IL-17-producing helper T (Th17) cells are a distinct T cell subset characterized by its pathological role in autoimmune diseases. Our group previously showed that Th17 cells function as osteoclastogenic helper T cells in bone destruction associated with inflammation, and that inhibition of Th17 development has the potential of a beneficial impact on bone diseases including rheumatoid arthritis (RA) [1]. It is therefore important to comprehend the molecular mechanism underlying Th17 development in order to develop ideal therapeutic strategies against RA.

IL-6 and TGF-β induce Th17 development, in which the orphan nuclear receptors RORγt and RORα play an indispensable role. We found that the expression of a nuclear IκB family member, IκBζ (encoded by the *Nfkbiz *gene), was upregulated by the combination of IL-6 and TGF-β, but independently of RORγt [2]. Not only *Nfkbiz*^-/- ^mice but also *Rag2*^-/- ^mice transferred with *Nfkbiz*^-/- ^CD4^+ ^T cells were highly resistant to experimental autoimmune encephalomyelitis, which is a mouse model of multiple sclerosis. *Nfkbiz*^-/- ^mice were also protected from the activation of osteoclastogenesis and bone destruction in a LPS-induced model of inflammatory bone destruction. When activated *in vitro *under Th17-polarizing conditions, IL-17 production in *Nfkbiz*^-/- ^T cells was markedly reduced compared to WT cells. Notably, the expression of RORγt and RORα was comparable between WT and *Nfkbiz*^-/- ^T cells. Thus, it is unlikely that ROR nuclear receptors function downstream of IκBζ or vice versa.

In the absence of IL-6 and TGF-β, neither the ROR nuclear receptors nor IκBζ induced Th17 development efficiently. However, when IκBζ was overexpressed, either RORγt or RORα strongly induced IL-17 production, even in the absence of exogenous polarizing cytokines. In cooperation with RORγt and RORα, IκBζ enhanced *Il17a *expression by directly binding to the regulatory region of the *Il17a *gene. In addition, the expression of *Il17f*, *Il21 *and *Il23r *mRNA was decreased in *Nfkbiz*^-/- ^T cells. IκBζ also bound to the promoter or the enhancer region of these genes in Th17 cells. Our study demonstrates the essential role of IκBζ in Th17 development, and points to a molecular basis for a novel therapeutic strategy against autoimmune disease.
